# The ovulatory and luteotropic actions of the male-derived beta-nerve growth factor in South American camelids

**DOI:** 10.1093/af/vfac037

**Published:** 2022-08-12

**Authors:** Luis Paiva, Mauricio Silva, Rodrigo Carrasco, Marcelo Héctor Ratto

**Affiliations:** Escuela de Medicina Veterinaria, Facultad de Agronomía e Ingeniería Forestal, Facultad de Ciencias Biológicas y Facultad de Medicina, Pontificia Universidad Católica de Chile, Santiago, Chile; Departamento de Medicina Veterinaria y Salud Pública, Núcleo de Investigación en Producción Agroalimentaria, Facultad de Recursos Naturales, Universidad Católica de Temuco, Temuco, Chile; Department of Chemistry, College of Arts and Science, University of Saskatchewan, Saskatoon, SK,Canada; Instituto de Ciencia Animal, Facultad de Ciencias Veterinarias, Universidad Austral de Chile, Valdivia, Chile

**Keywords:** alpaca, biotechnology, llama, ovulation-inducing factor, reproduction

**Implications**

- The neurotrophin beta-nerve growth factor (**β-NGF**) has a potent LH-releasing effect and is the main trigger for ovulation in camelids.
- The β-NGF exerts luteotropic effects in camelids not only via enhanced LH release but also by acting at the ovary, where stimulates steroidogenic enzyme expression in luteal cells as well as vascularization of the corpora lutea.
- The mechanism of ovulation of β-NGF implicates central neuroendocrine systems that result in GnRH release.
- Also, recent works have shown pro-luteotropic effects of β-NGF in cattle. Thus, β-NGF might be a potential biotechnology tool to enhance fertility in farm production systems.

## Introduction

Induced ovulation is considered an evolutionary reproductive trait developed by some species to achieve fertilization success (Bakker and Baum, 2000). This ovulation mechanism is mostly observed in animal species exhibiting low population densities or inhabiting highly seasonal environments, and it is intimately linked to copulation (Bakker and Baum, 2000). Tactile, visual, and olfactory stimuli occurring during mating behavior have been classically linked to eliciting or facilitating ovulation in induced ovulators. Since its early definition, the mechanical stimulation of the vagina and cervix during penile intromission that stimulates afferent sensory inputs has been ascribed to the main factor triggering a neuroendocrine reflex responsible for the preovulatory luteinizing hormone (LH) surge and subsequently ovulation (Kauffman and Rissman, 2006). However, not much consideration has been given to the chemical or hormonal effect that semen can have on triggering the release of the oocyte.

The notion that the male could signal and influence the female reproductive physiology to improve the conditions for pregnancy success through semen deposition in the female tract is a notion just recently considered (Robertson, 2007). In fact, several studies have demonstrated that molecules present in the seminal fluid of different species can affect fertilization, early embryo survival, endometrial receptivity, and finally pregnancy outcome (Robertson, 2007). Recently, it has been proposed to extend further the concept of pheromones, based on the observations made in camelids, to include along with the classic air-borne chemical signals a separate class of seminal molecules that act on the female reproductive tract (Robertson and Martin, 2022).

**Figure 1.**
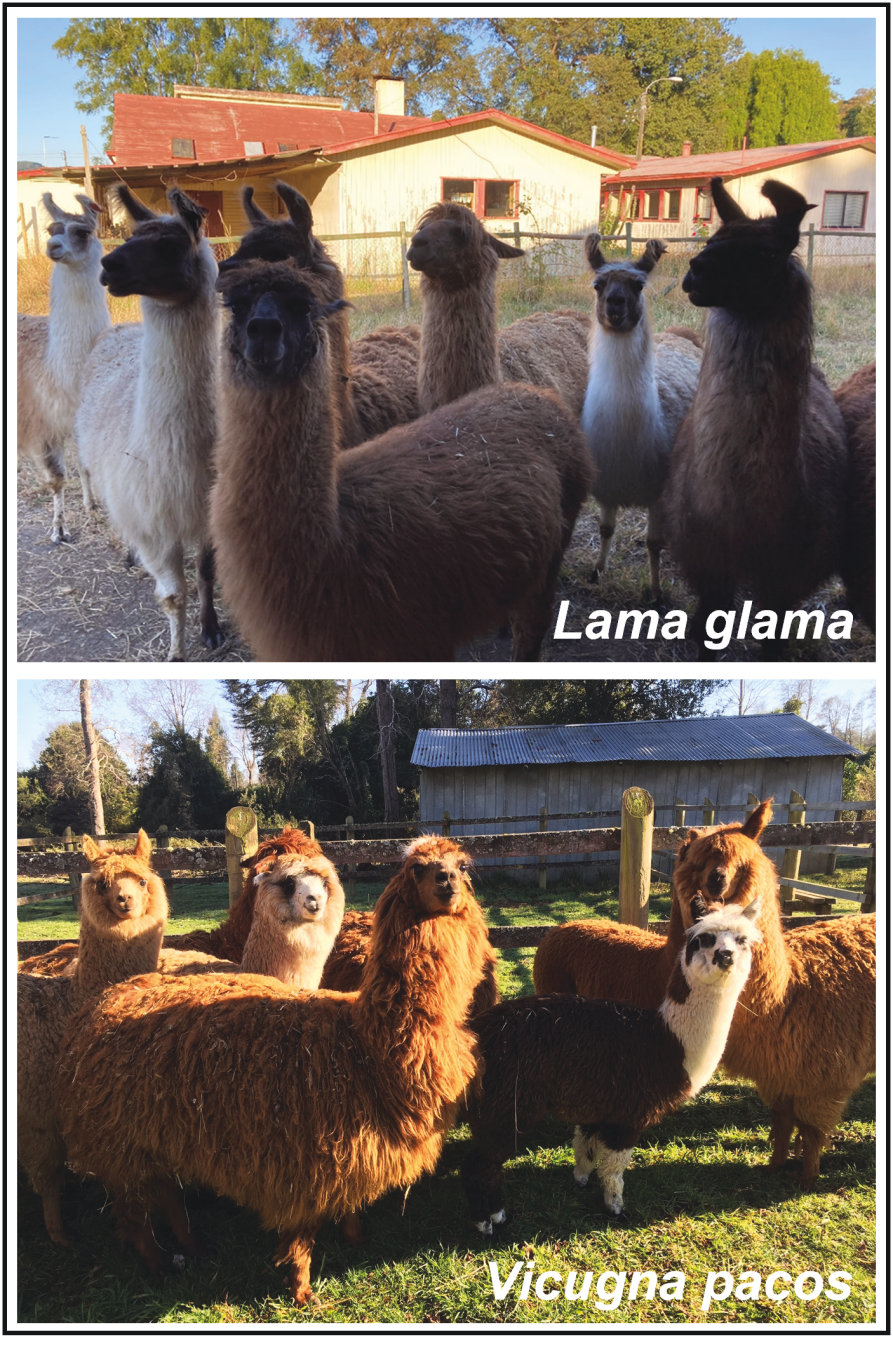
Domestic South American camelids. Llamas (upper panel) and alpacas (low panel) are domestic species native to the Andean region of Peru, Bolivia, and northern Chile and Argentina. Small herds can be easily kept for different purposes in lowlands as illustrated in the pictures, which are located in Southern Chile.

Seminal plasma is the largest portion of an ejaculate containing sugars, salts, lipids, proteins, hormones, antimicrobial molecules, and others acting as immunity suppressors. Nowadays, it is clear that apart from the role of several of these molecules on sperm physiology, they have a role in chemical communication between males and females that surpass mating itself (Robertson, 2007; Schjenken and Robertson, 2020). In this regard, findings reported during the last 30 yr provide robust evidence of the presence of an ovulation-inducing factor (**OIF**) in the seminal plasma of old (Bactrian and Dromedary camels) and new (llamas and alpacas; Figure 1) world camelids, which unequivocally support a pivotal role of the male on the control of female reproduction via seminal plasma signals in camelids.

Early South American studies classified female llamas and alpacas as induced ovulators based on experimental designs that included different mating variations (San-Martin et al., 1968; Fernandez-Baca et al., 1970). These studies established the classic notion that the mechanical stimulation occurring during copulation was required to induce the preovulatory LH surge and subsequent ovulation. However, as mentioned, later studies elucidated and provided depth insights into the mechanism of induced ovulation in domestic South American camelids, which is driven by a protein factor present in the seminal plasma rather than by physical stimulation of the female genital tract and subsequent nerve transduction (Ratto et al., 2012). Besides the effect on the induction of ovulation in llamas and alpacas that occurs shortly after systemic protein administration, this ovulation-inducing factor also enhances the development and function of the corpus luteum (Adams et al., 2005; Silva et al., 2014; Ulloa-Leal et al., 2014), a potential strategy to stimulate early embryo development and implantation.

The present review aims to condense most of our current understanding regarding the major female reproductive effects of the OIF—also known as beta-nerve growth factor (**β-NGF**)—that is present in the male llama and alpaca seminal plasma, as well as its possible mechanisms of actions in the brain which still awaits to be elucidated. A potential use of β-NGF in the light of recent evidence of its effect in cattle is also reviewed.

## A Brief History of the Discovery, Characterization, and Identification of OIF

In the ‘80s, the pioneering work of Chinese researchers (Chen et al., 1983) brought to our attention the fact that a high rate of ovulations was induced after intravaginal deposition of semen in Bactrian camels, an induced ovulation species. After two decades of intense work, the research conducted in Bactrian camels has led to the following findings: 1) rather than the mechanical stimulation of copulation, a protein present in the seminal plasma is responsible for triggering ovulation in this species (Chen et al., 1983, 1985); 2) after systemic or intrauterine administration of the seminal factor, a rapid preovulatory LH surge is induced (Xu et al., 1985); 3) the protein factor is chemically different from other ovulatory molecules (gonadotropin-releasing hormone [GnRH], LH, Human chorionic gonadotropin [hCG], equine chorionic gonadotropin [eCG], and prostaglandin F2alpha [PGF2α] ; Pan et al., 2001); and 4) this factor is transported via the bloodstream to upper central structures that control reproduction (Zhao et al., 1990). However, the complete identification of the seminal factor in Bactrian camels could not be accomplished, so generically it was called OIF.

Two decades after the Bactrian camel studies, Adams et al. (2005) reported the existence of an OIF in the seminal plasma of llamas and alpacas, demonstrating that the administration of a single intramuscular dose of seminal plasma can lead to high rates of ovulation and also an enhanced preovulatory LH surge that, in turn, determined a larger secretion of progesterone from the resulting corpus luteum, and so providing the first evidence of a luteotropic effect of OIF.

Since these early studies, llamas and alpacas have constituted a useful animal model to study the OIF using either homologous or heterologous seminal plasma (Adams et al., 2005; Ratto et al., 2005, 2006), seminal fractions (Ratto et al., 2010, 2011), or purified OIF (Silva et al., 2011; Tanco et al., 2011; Ulloa-Leal et al., 2014) given by intramuscular, intravenous, or intrauterine routes. In the last decade, this factor has been biochemical and functionally characterized in llamas and alpacas as the neurotrophin β-NGF (Ratto et al., 2011, 2012, Kershaw-Young et al., 2012), a molecule exhibiting a potent ovulatory effect in both alpacas and llamas, and also a significant luteotropic effect in llamas (Adams et al., 2005; Silva et al., 2014; Ulloa-Leal et al., 2014). Nevertheless, a similar effect has not been confirmed in alpacas (Kershaw-Young et al., 2012; Stuart et al., 2015). Subsequent studies also reported its presence in the seminal plasma of other species, including dromedary camels (Druart et al., 2013; Kumar et al., 2013).

**Figure 2.**
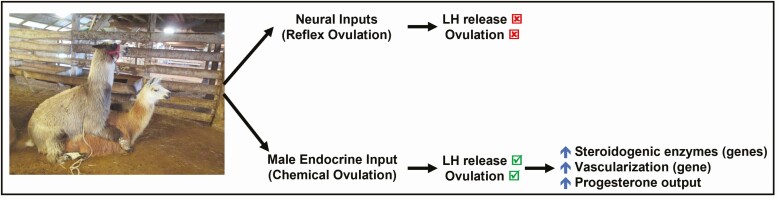
Chemically induced ovulation in South American camelids. Female llamas mating with an urethrostomized male completely failed to ovulate, whereas mating with an intact male or administration of β-NGF induces LH release that triggers ovulation. Furthermore, corpus luteum induced with β-NGF, but not GnRH, shows enhanced expression of enzymes involved in not only steroid synthesis but also vascularization, yielding high progesterone input.

In a cleverly designed experiment, Berland et al. (2016) confirmed that intrauterine administration of seminal plasma, but not penile intromission, is sufficient to elicit the LH surge and ovulation in this species: female llamas mated with an intact male or given an intrauterine infusion of seminal plasma showed 86% and 83% ovulation rate, respectively, while females mated with an urethrostomized male llama completely failed to ovulate (0%; Figure 2). Also, the circulating concentration of LH was positively correlated with an increase in plasma β-NGF in the same study. This LH surge induced by systemic administration of purified llama β-NGF can be abolished in llamas pretreated systemically with the GnRH antagonist, cetrorelix, which blocks GnRH membrane receptors at the gonadotrophs (Silva et al., 2011). These pieces of evidence suggest that seminal β-NGF is absorbed through the endometrium following copulation and, consequently, entering systemic circulation to stimulate GnRH release by direct or indirect actions on hypothalamic GnRH neurons, eliciting the preovulatory LH surge. This mechanism represents a whole new category of induced ovulation, which is chemically, but not physically, induced (Adams et al. 2016; Silva et al. 2020).

## Neurotrophins and Reproductive Function

The family of neurotrophins regulates the development and maintenance of peripheral sympathetic and sensory neurons, as well as some cholinergic neurons. This family includes nerve growth factor (NGF), brain-derived neurotrophic factor (BDNF), neurotrophin 3 (NT-3), neurotrophin 4 (NT-4), neurotrophin 5 (NT-5), and neurotrophin 6 (NT-6), the latter present only in teleost fish (Pezet and McMahon, 2006). All these neurotrophins bind the low-affinity transmembrane receptor known as p75^NTR^, but each neurotrophin binds with high affinity to its own specific Tyrosine kinases (Trk) receptor, which in the case of β-NGF corresponds to TrkA (Pezet and McMahon, 2006). Nowadays, it is known that, in addition to the nervous system, the actions of NGF are also exerted in other tissues, including testes and ovaries (Ayer-LeLievre et al., 1988; Lara et al., 1990; Dissen et al., 2001; Jin et al., 2006), indicating a potential role in sperm and follicular physiology.

### The β-NGF as a neurosecretory trigger

Since the initial report of Adams et al. (2005), there has been an intense search for the site of action of β-NGF camelids. Early in vitro studies in llama, cow, and rat pituitary cells showed that either purified β-NGF or alpaca seminal plasma led to LH secretion in the culture media suggesting a direct effect on the pituitary (Paolicchi et al., 1999; Bogle et al., 2012). However, in vivo pharmacological blockade of the GnRH receptor in llamas completely prevents LH release and ovulation in response to β-NGF (Silva et al., 2011). Given that hypothalamic GnRH secretion is the main trigger for pituitary LH secretion (Clarke and Cummins, 1982), it has been hypothesized that the β-NGF-mediated mechanism is guided directly or indirectly by the GnRH system in the hypothalamus.

In the last decades, the view that has dominated the neuroendocrine field is that GnRH neurons operate under the influence of afferent neurons that enhance or suppress the hypothalamic GnRH output (transsynaptic mechanism; Ojeda et al., 2003). This concept is derived from studies conducted in spontaneous ovulatory species where GnRH neurons do not contain the relevant estrogen receptor for an LH surge (Shivers et al., 1983, Herbison and Theodosis, 1992, Lehman and Karsch, 1993). This notion was evaluated by testing whether NGF receptors are expressed in GnRH neurons of llama, showing low colocalization of p75^NTR^ and TrkA in GnRH neurons (0% and 2.5%, respectively; Carrasco et al., 2018), which suggest that β-NGF may be controlling GnRH output by interacting with an interneuron in the hypothalamus. In a subsequent study, several populations of neurons colocalizing TrkA and p75^NTR^ receptors were found in the forebrain, single-labeled TrkA neurons were found in the periventricular hypothalamus, and also ependymal cells expressing p75^NTR^ were found in the third ventricle (Carrasco et al., 2021b).

A recent study, which examined the activation of GnRH neurons after intravenous β-NGF administration, did not show differences in the proportion of GnRH neurons expressing Fos protein (a well-established marker of neuronal activation; Moenter et al., 1993) after 4 h when compared with saline-treated animals, although LH concentrations were 3-fold higher in β-NGF-treated llamas (Carrasco et al., 2021a). The role of progesterone (an inhibitor of GnRH release; Skinner et al., 1998) on the effects of β-NGF in llamas was also evaluated, revealing that circulating concentrations as high as 14 ng/mL failed to impair the β-NGF-induced LH surge (Carrasco et al. 2021a).

The kisspeptin system and the role of its peptide product, a well-known secretagogue of GnRH in spontaneous ovulators, have been assessed for its potential involvement in β-NGF-induced ovulation in llamas (Carrasco et al., 2020; Berland et al., 2021); NGF receptors were not identified in kisspeptin neurons of both the preoptic area and the arcuate nucleus; however, systemic administration of kisspeptin led to an increase of LH release (Carrasco et al., 2020). Since the morphological evidence does not favor a transsynaptic route of action for β-NGF, it could be that β-NGF acts on a different mechanism that perhaps does not directly involve neuronal elements in the hypothalamus.

**Figure 3.**
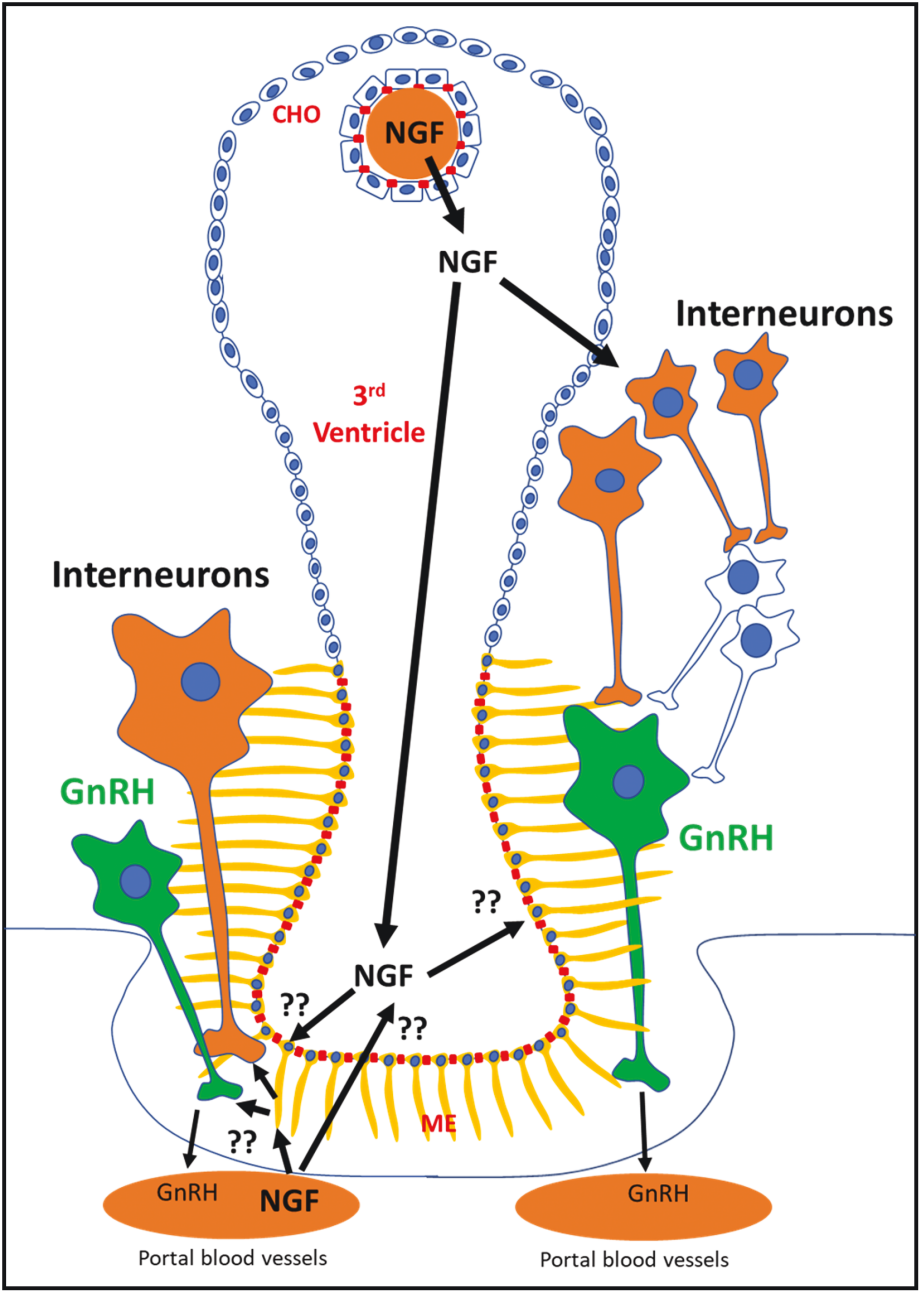
Potential mechanisms of action of β-NGF to trigger the preovulatory GnRH release in llamas. Systemic β-NGF may act by penetrating the brain at choroid plexuses (CHO)/blood–brain barrier to act on β-NGF-responsive neurons that activate GnRH neurons and/or by acting on tanycytes expressing p75^NTR^, promoting their retraction that allows the release of the GnRH peptide into the portal blood vessels.

If systemic β-NGF does not penetrate into the brain to elicit its actions, a plausible explanation of the effects of systemic β-NGF on GnRH release could be that it acts on targets located outside of the blood–brain barrier, such as the circumventricular organs. An emerging body of research has shown an important role of tanycytes in regulating neurosecretory processes in the median eminence, a circumventricular organ. Tanycytes are a group of elongated ependymal cells that are part of the blood–brain barrier where they are in contact with both portal blood and Cerebrospinal fluid. In rodents, the association of tanycytes and GnRH terminals undergoes constant plastic changes in accordance with the estrous cycle (Reviewed by Prevot, 2002). The presence of p75^NTR^ in tanycytes of llama median eminence has been recently identified (Carrasco et al., 2020), similarly as in primates (Borson et al., 1994; Blurton-Jones et al., 1999) and rodents (Yan and Johnson, 1989; Pioro and Cuello, 1990; Koh and Higgins, 1991) studies; in the latter species, a recent study has reported a close association between GnRH fibers and p75^NTR^-expressing tanycytes (Pinet-Charvet et al., 2020). It is puzzling that the low-affinity receptor of β-NGF is expressed in tanycytes because most biological effects of β-NGF known to date are mediated by the TrkA receptor. Currently, it is unclear whether tanycytes are the target of β-NGF; however, their key location at the median eminence—where GnRH is released—offers a potential explanation for the fast response in LH concentrations after β-NGF administration (~15 min; Figure 3).

### The luteotropic effect of β-NGF

In addition to the well-established ovulatory effect of β-NGF mediated by the release of the preovulatory LH surge from the pituitary gland that is determinant for the initial stage of luteinization, and corpus luteum formation and function, the administration of β-NGF also exerts a potent luteotropic effect in llamas as reported in several studies (Adams et al., 2005; Tanco et al., 2011; Ulloa-Leal et al., 2014). The formation of the corpus luteum by intrauterine infusion or intramuscular administration of β-NGF consistently results in higher progesterone output from the early stages of corpus luteum development than those induced after GnRH administration. Moreover, it has been established a positive relationship between the magnitude of the LH peak and the following luteal function when females are treated with either β-NGF purified from seminal plasma or whole seminal plasma (Tanco et al., 2011; Silva et al., 2015). These pieces of evidence have served as a substrate to hypothesize that the secretory pattern of LH induced by β-NGF is the main responsible event for the luteotropic effect in llamas.

**Figure 4.**
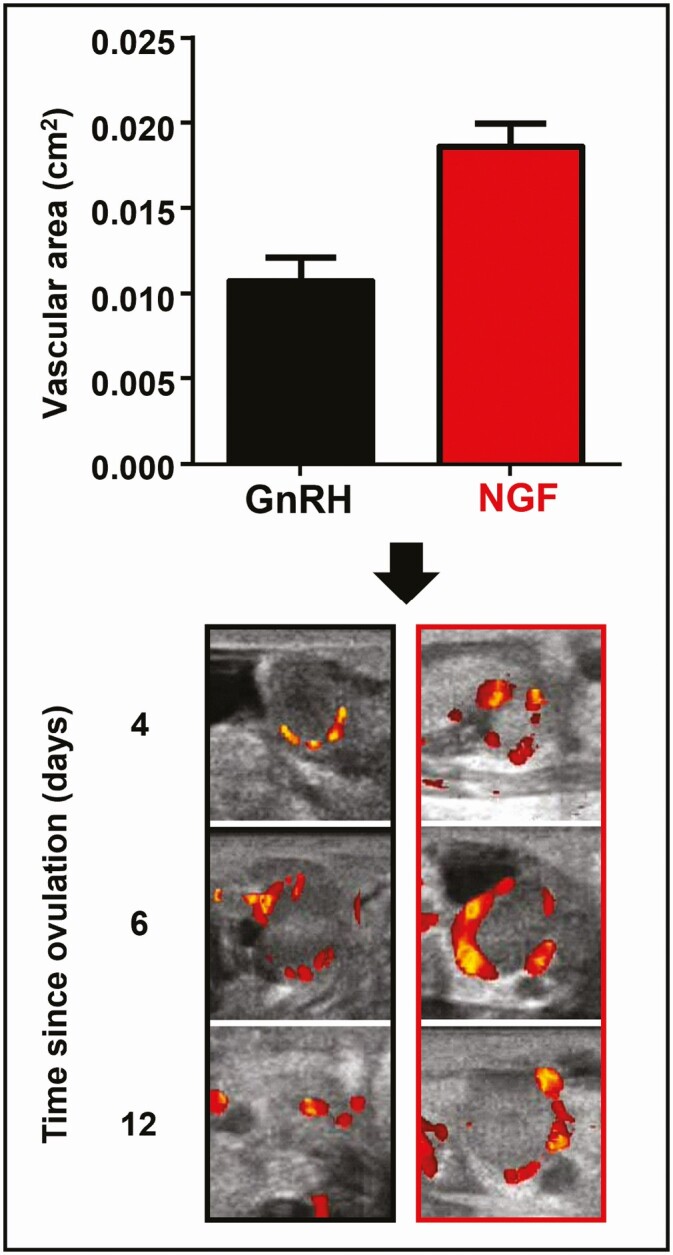
Effect of β-NGF on the vascularization of corpus luteum. Ovulations induced by administration of β-NGF lead to an increased vascular area of the resulting corpus luteum than the ones induced by GnRH.

Follow-up ultrasonography studies have shed light on the luteotropic effect of β-NGF. Power Doppler ultrasonography, a technique that provides detail of blood flow, has shown that both the preovulatory follicle and the early corpus luteum exhibit greater vascularization in llamas treated with seminal plasma β-NGF than those injected with GnRH (Figure 4), and this results in higher levels of progesterone in the blood (Ulloa-Leal et al., 2014; Silva et al., 2017). This increase in vascularization has also been corroborated in a subsequent histological analysis (Silva et al., 2017). Furthermore, vascular endothelial growth factor (VEGF), a molecule that induces angiogenesis by stimulating the proliferation of endothelial cells of preexisting capillaries (Dvorak et al., 1999), seems to be key to eliciting the luteotropic effect in vivo as administration of β-NGF leads to enhanced expression of the gene that codifies for this protein factor (Valderrama et al., 2019).

Interestingly, evidence originating from different spontaneous and induced ovulation species indicates that follicles are equipped with the machinery to bind and transduce β-NGF actions: expression of both TrkA and p75^NTR^ NGF receptors in granulosa and theca cells has been found in rabbits (Maranesi et al., 2018), cows (Dissen et al., 2000), rats (Dissen et al., 1996), and humans (Salas et al., 2006); these pieces of evidence suggest that the luteotropic action of β-NGF may also be exerted directly at the ovary. Consistent with this notion, the application of β-NGF to granulosa cells collected from llama preovulatory follicles results not only in enhanced gene expression of the vascular factor VEGF but also in the expression of steroidogenic enzymes involved in progesterone synthesis and the output of progesterone hormone (Valderrama et al., 2020). Moreover, this in vitro effect has been shown to result in higher upregulation of the expression of VEGF and STAR genes after 20 h than the observed in cells treated with LH (Valderrama et al., 2019). Finally, an in vivo llama study (Fernández et al., 2014) shows that multiple administration of β-NGF during the periovulatory stage induces greater luteal vascularization and progesterone secretion than that observed in those females treated with a single dose.

In addition to the effect on vascular development, female llamas treated with seminal plasma β-NGF exhibit the upregulation of genes encoding for enzymes involved in steroid synthesis in luteal tissue, including the p450_SCC_ and STAR on days 4 and 8 of the luteal phase, which results in enhanced progesterone secretion (Silva et al., 2017). Moreover, both natural mating and systemic administration of purified llama β-NGF have been reported to induce a rapid shift from estradiol to progesterone synthesis (an indicator of luteinization) in the preovulatory follicle in llamas: the upregulation of the main genes related to progesterone production results in an increased progesterone/estradiol ratio in the follicular fluid (Valderrama et al., 2019). This in vivo effect on gene expression and progesterone production is greater in llamas given with β-NGF or submitted to mating than those given with GnRH alone, indicating a direct effect on the ovary (Silva et al., 2017; Valderrama et al., 2019).

## Perspective: Is the β-NGF a Potential Biotechnological Tool in Farm Animals?

Studies conducted in cattle, a species of spontaneous ovulation, show that the administration of β-NGF purified from llamas does not induce ovulation in pre-pubertal heifers, but it does have a luteotropic effect in sexually mature heifers and accelerates the appearance of the next follicular wave when β-NGF is intramuscularly given during the first follicular wave (Tanco et al., 2012). In another bovine study, Tribulo et al. (2015) did not observe an increase in LH concentration or ovulation when heifers were intramuscularly given with a volume of 12 mL of bovine seminal plasma containing at least 250 µg of β-NGF, but a more rapid increase in progesterone secretion and a longer corpus luteum lifespan was observed. In this sense, Carrasco et al. (2016) observed abundant expression of the high-affinity receptor TrkA, in antral ovarian follicles and in the corpus luteum throughout the estrous cycle in cattle. Furthermore, in vivo and in vitro gene expression of TrkA receptors in rat preovulatory follicles is reported to be elicited by the progressive discharge of LH (Dissen et al. 1996), indicating a potential role of LH in the regulation of the expression of NGF receptors in follicular and luteal cells. A recent study in bovine (Stewart et al., 2020) analyzed the effect of β-NGF in heifers when this is incorporated in an estrus synchronization protocol using GnRH; heifers treated with GnRH plus β-NGF exhibited enhanced LH release and an LH peak 2-fold higher than those heifers receiving only GnRH. Similarly, a significant increase in luteal vascularization and progesterone secretion in heifers treated with a dose of 1 mg of purified llama β-NGF during the preovulatory LH surge induced by a progesterone/estradiol-based estrus synchronization protocol was detected when compared with their control non-treated counterparts (Gajardo et al., 2021). This evidence indicates that β-NGF could exert a luteotropic effect in cattle not only by increasing LH release but also by acting at the ovarian level.

## Conclusions

The molecule β-NGF that is present in the male seminal plasma of llamas and alpacas has been shown to be essential for reproduction in these species. Evidence indicates that the sole administration of β-NGF is sufficient to elicit a strong ovulatory and luteotropic effect in these species, challenging our conception of induced ovulation in mammals. However, the central mechanism by which β-NGF stimulates the preovulatory release of GnRH that, in turn, stimulates the release of LH that causes ovulation has yet to be elucidated. Finally, recent studies conducted in cattle indicate that the luteotropic effect of β-NGF is conserved in this species, thereby opening a new avenue for the use of this molecule as a potential tool to enhance fertility in farm production systems.

## About the Authors

**Luis Paiva** has been recently appointed as an Assistant Professor in Animal Physiology in the School of Veterinary Medicine at the Pontificia Universidad Católica de Chile. He received his DVM from the Universidad Católica de Temuco and an MSc in Cell Biology from the Universidad Austral de Chile. He then gained his PhD (Integrative Physiology) from the University of Edinburgh, UK. He is interested in understating how peripheral inputs modulate the activity and release of neuroendocrine cells, which then modulate other pivotal physiological functions. His research has focused on the physiology of oxytocin and GnRH systems in laboratory and domestic animals.

**Mauricio Silva** is a professor and researcher in Animal Reproduction at the Universidad Católica de Temuco, Chile. His background encompasses a DVM degree, an MSc in Animal Reproduction, and a PhD in Veterinary Sciences, all received from the Universidad Austral de Chile. His research interests are reproductive physiology and biotechnologies in ruminants and camelids, with an emphasis on in vitro embryo production in cattle and camelids. During the last 10 yr, he has collaborated with Prof. Marcelo Ratto to unveil the mechanism of ovulation in llamas. Since the discovery of the ovulation-inducing factor in the seminal plasma of llamas and alpacas, he has investigated not only how this molecule interacts with the female central nervous system to trigger ovulation but also its luteotropic effect.

**Rodrigo Carrasco** received his D.V.M. degree from Universidad Austral de Chile in 2012 (Valdivia, Chile). For his graduate work, he attended the University of Saskatchewan (Saskatoon, Canada) where he obtained an M.Sc. and a Ph.D. in Veterinary Biomedical Sciences. Currently, he is a Postdoctoral fellow at the Department of Chemistry of the University of Saskatchewan. His research interests are the neuroendocrine control of hormonal secretion, GnRH neuronal function, and the neural control of gonadal function.

**Marcelo Héctor Ratto** is a Full Professor in the Institute of Animal Science, Faculty of Veterinary Sciences at the Universidad Austral de Chile, where he teaches reproductive physiology and biotechnology in undergraduate and graduate courses. He obtained his DVM from the Universidad Nacional de la Plata, Argentina, an MSc in Animal Reproduction from the Universidad Austral de Chile, and a PhD from the University of Saskatchewan, Canada. He conducts basic and applied research in llamas and ruminants (cows and sheep) investigating the effect of the neurotrophin β-NGF on ovarian and uterine function. He is also interested in incorporating this factor in estrus synchronization protocols for fixed-time artificial insemination in dairy cattle to improve fertility.
